# The Impact of Foreign Direct Investment on Environmental Pollution in China: Corruption Matters

**DOI:** 10.3390/ijerph17186477

**Published:** 2020-09-05

**Authors:** Shi Wang, Hua Wang, Qian Sun

**Affiliations:** 1 School of Economics and Finance, Xi’an International Studies University, Xi’an 710128, China; wangshi1107@xisu.edu.cn; 2 Institute of Communication and Global Public Opinion, Xi’an International Studies University, Xi’an 710128, China; 3 “One Belt One Road” Economic and Trade Cooperation Innovation Team, Xi’an International Studies University, Xi’an 710128, China; 4 School of Foreign Studies, Xi’an Jiaotong University, Xi’an 710049, China; 5 School of Economics and Management, Southwest Petroleum University, Chengdu 610500, China; sunqianinhk@outlook.com

**Keywords:** FDI, corruption, environmental pollution

## Abstract

This research investigates the interaction effect between corruption and foreign direct investment (FDI) on environmental pollution by applying the spatial econometric model to the panel data of China’s 29 provinces from 1994 to 2015 and analyzes the differences between China’s eastern, central and western regions. Results show that (a) FDI inflow deteriorates the environmental quality, validating the pollution haven hypothesis (PHH); (b) by weakening the environmental standards, corruption enables the inflow of low-quality FDI, weakens the spillover effect of FDI and indirectly causes further environmental pollution; (c) the interaction effect between corruption and FDI on environmental pollution is less significant in the eastern region than in the central and western regions.

## 1. Introduction

FDI inflow into China has been increasing since the reform and opening-up, a growth from the actually utilized FDI of a mere 1.96 billion US dollars in 1985 to 134.97 billion US dollars in 2018, a 69-times increase or 20% annual growth. FDI has been a major propeller of China’s rapid economic growth in recent years. However, an accompanying side effect of the reform, opening-up and FDI inflow is the rampant environmental pollution. Specifically, the environmental pollution caused by FDI inflow is called the “pollution haven” phenomenon. This is because some enterprises from developed countries cannot fulfill the stringent environmental regulations and seek large-scale cost reduction by adopting high-pollution technologies in developing countries. Therefore, FDI flows into developing countries with low environmental standards or even no environmental regulations and causes pollution problems. It should be noted that, when corruption is less rampant among government officials in a developing country, environmental regulations can be sufficiently carried out; thus, FDI usually fulfills the corresponding regulations of the host country. On the contrary, if corruption is more serious among the officials, low-quality and environmental unfriendly FDI could enter the country by bribing, further exacerbating the environmental pollution, i.e., the vicious circle between corruption and pollution caused by FDI. The aforementioned analysis indicates that FDI may be the main culprit of pollution emissions, including chemical oxygen demand (COD) in industrial wastewater, sulfur dioxide and nitrogen oxides in industrial waste gas, industrial soot and dust. In addition, the rampant corruption among host country government officials may be a catalyst.

This paper is closely related to the literature about how FDI affects environmental pollution in host countries. Research in this field mainly includes three viewpoints: the first is the pollution haven hypothesis, which asserts that the high-polluting enterprises in developed countries transfer to the developing countries with relatively low environmental regulation standards and pollution costs, which leads to environmental degradation in the host country [1,2,3,4,5,6,7]. The second one is the pollution halo hypothesis, which argues that FDI provides an opportunity for developing countries to adopt new technologies to enable clean or green production and improves the environmental quality of the host country [8,9,10]. The third viewpoint holds that the influence of FDI on environmental pollution is the comprehensive result of multiple mechanisms [11,12]. However, these studies do not take corruption into consideration. The analysis above suggests that, in developing countries with high levels of corruption, such as China, the FDI–emissions nexus is likely to be affected by official corruption. If corruption is ignored, the environmental pollution effect of FDI may be misestimated. This paper will study this issue by applying the spatial econometric model to the panel data of China’s provinces.

The rest of this study is structured as follows. Section 2 reviews the literature on FDI, corruption and environmental pollution. Section 3 describes the specification of the econometric model, definitions of variables and data sources. Section 4 presents the results and discussion of empirical analysis in detail. Section 5 concludes and Section 6 puts forward possible policy implications.

## 2. Literature Review

### 2.1. Institutional Quality and Corruption

The existing literature shows that the quality of the political institution is an important factor affecting the level of corruption in a country. Based on data from 64 developing countries between 1984 and 2004, Yadav [13] found that corruption was closely linked to two institutional arrangements: one was that the political parties could control the agenda of parliament; the other was that the political parties could purge the members of the opposition from the party. Pellegata [14] analyzed data from more than 100 countries from two dimensions: the level of democracy and the length of the period of democratization. The results showed that the development of democratization would significantly reduce the level of corruption in these countries. However, Rock [15] believed that the relationship between democratization and corruption was not linear but an inverted U-shaped relationship: in the early stage of democratization, the democratic reform made the corruption information between corrupt officials and bribers more sufficient, thus exacerbating the corruption. The deepening of democratization increased the punishment for corruption, which raised the cost of corruption and reduced corruption. Using data for 82 countries, Kotera et al. [16] found that, in countries with a high degree of democratization, the increase in government size reduced corruption, and vice versa. The reason was that countries with a low degree of democratization lacked the effective supervision mechanism for their governments, so the expansion of government scale aggravated corruption. When the level of democratization reached a high level, the supervision mechanism of the government was relatively perfect, which could restrain the corrupt behavior of officials.

### 2.2. FDI and Environmental Pollution

Extant literature has well documented the connection between FDI and the host country’s environmental quality with three main viewpoints. The first is the pollution haven hypothesis (PHH), i.e., corporations from developed countries transfer high-pollution factories to developing countries with low environmental regulations in the form of FDI to avoid the stringent regulations and high costs in their domestic countries, causing the rapid deterioration of environmental quality of the host developing countries. First proposed by Walter and Ugelow [1], the PHH was further elaborated by Levinson and Taylor [17] with a global value chain and division of labor explanation; that is, developing countries undertake certain roles in the value chain, and corporations from developed countries transfer high-pollution industries into these developing countries under the name of division of labor. For example, based on panel data of China’s industries from 2000 to 2010 with a two-step generalized method of moments (GMM), Ren et al. [18] analyzed the impact of international trade and FDI on environmental pollution and found that both factors were positively associated with carbon dioxide emissions in China.

In addition, with time series data for the period of 1980 to 2010 for 14 Latin American countries and panel fixed and random effects models, Sapkota and Bastola [19] found that FDI inflow led to environmental degradation in these countries, even when the effects of physical capital, energy, human capital, population density, and unemployment rate were controlled. Solarin and Al-Mulali [20] analyzed the effect of FDI on carbon dioxide emissions, carbon footprint, and ecological footprint for 20 countries and found that FDI increased pollution in the developing countries. With panel data of 25 emerging markets in the Asian region from 1980 to 2016 and panel cointegration fully modified ordinary least squares (FMOLS), To et al. [21] found that the FDI inflows significantly worsened the environmental quality of these countries. For other recent research, see Mert et al. [22], Ma et al. [23], and Sarkodie and Strezov [24].

The second viewpoint holds that FDI inflow does not worsen environmental quality. On the contrary, it helps to improve the environment, the most representative theory being the pollution halo hypothesis, i.e., FDI usually possesses more advanced and cleaner production and emission technologies than those of the local firms in the host developing countries. A technology spillover may also occur when multinational corporation employees transfer to domestic corporations. Moreover, FDI corporations increase the competition level in the market, forcing domestic corporations to increase their research and development or directly imitate the production and management methods of the FDI corporations, a phenomenon called the horizontal technology spillover effect [25]. Multinational corporations may only be willing to procure raw materials and intermediate goods from local corporations that fulfill environmental regulations. Such interactions in the supply chain enable FDI to improve the overall technological level with forward and backward vertical technological spillover effects and improve the efficiency and cleanliness of production methods. Therefore, FDI inflow facilitates environmental quality improvements in developing countries [26]. Based on the spatial Dubin model, Huang et al. found that FDI inflow in China brought positive effects on both economic growth and environmental quality, and the effects were even more significant by FDI from Hong Kong, Macau, and Taiwan than by FDI from other non-ethnic Chinese countries and regions [9].

With panel data of 46 sub-Saharan African countries for the period 1980–2015 and fixed and random effect estimation techniques, Acheampong et al. [27] found that foreign direct investment reduced carbon emissions while trade openness deteriorated the environment. Ayamba et al. [28] found that foreign direct investment reduced carbon emissions, using panel data of 31 provinces in China from 1995 to 2016 and the simultaneous equation method. They advised that China should encourage FDI with advanced production technology. For other recent research, see Jiao et al. [29], Ning and Wang [30], Shao et al. [31], Cheng et al. [32], and Khan et al. [33].

The third viewpoint is that the impact of FDI on environmental pollution is a complex mechanism; that is, FDI affects environmental quality through the scale, structure, and technology of the corporations, and the aggregate effect of these factors is the ultimate effect of FDI on the environment. Previous studies derived contradictory results on the direction and consequences of these effects. With a simultaneous equation model, He [34] found that FDI increased pollution through the scale and structure effect but decreased pollution through the technology effect. At the aggregate level, 1% more FDI induced 0.1% more industrial sulfur dioxide emission, i.e., a positive association between FDI and pollution. Assuming that FDI increased pollution through the scale effect but decreased pollution through the technology and regulation effects, Yan and An [35] found with China’s provincial panel data that FDI had an inverted U-shaped influence on sulfur dioxide emission but an N-shaped influence on nitrogen dioxide emission.

With panel data of 285 Chinese cities from 2003 to 2014 and global and local measures of spatial autocorrelation, Liu et al. [11] found that FDI did not necessarily lead to environmental degradation. Specifically, they found that FDI reduced waste soot and dust and increased wastewater and sulfur dioxide. Shahbaz et al. [12] analyzed the relationship between FDI and carbon emissions for the Middle East and North African (MENA) region for the period 1990–2015. They found that there was an N-shaped association between FDI and carbon emissions. Balsalobre-Lorente et al. [36] found an inverted U relationship between FDI and the ecological footprint, using a panel data model for four countries from 1990 to 2013.

### 2.3. Corruption and FDI

Previous research related to the effect of corruption on FDI includes two main branches. The first branch is the direction of the effect of corruption on FDI, which includes two viewpoints. One is the “sand the wheels” hypothesis, which asserts that corruption reduces the FDI inflow. Habib and Zurawicki [37] conducted a study with panel data on the association between host country corruption and FDI and found that host country corruption had a significant negative effect on FDI. Lambsdorff [38] analyzed the data of 54 countries and believed that corruption would hinder FDI inflow. Based on the data of FDI which flows from 12 developed countries to 45 host countries, Wei [39] also found that corruption had a negative impact on FDI inflow. Based on the data of 106 FDI host countries, Cuervo-Cazurra [40] further demonstrated that corruption had a negative impact on FDI. He also found that investors from countries that had signed the OECD Convention on Combating Bribery of Foreign Public Officials in International Transactions were more vulnerable to corruption than those from countries with a high level of corruption. Based on the data of 117 countries from 1984 to 2004, Al-Sadig [41] found that the level of corruption in the host country had a negative impact on FDI inflow, but the quality of the institution was more important than the level of corruption in attracting FDI inflow. Using the investment data of 6288 American multinational enterprises in China from 1993 to 2001, Du et al. [42] found that American multinational enterprises preferred to invest in areas with low levels of corruption, good intellectual property protection, low degrees of government intervention, and high contract efficiency. With panel data of East and Southeast Asian countries from 1995 to 2011, Quazi [43] found that corruption significantly impeded FDI, supporting the “grabbing hand” hypothesis. Recent studies such as such as those of Hakimi and Hamdi [44], Kasasbeh et al. [45], Manu and Patel [46], Belgibayeva and Plekhanov [47], and Karim et al. [48] have found similar results with different data sets and econometric methods.

The other is the “grease the wheels” hypothesis, which asserts that corruption facilitates FDI inflow. Hines [49] found that high-growth corrupt countries had a higher growth rate of FDI inflow than high-growth incorruptible countries. By analyzing the data of 73 developed and less developed countries from 1995 to 1999, Egger and Winner [50] found that corruption could stimulate FDI inflow. Moreover, the effect was significantly positive in both the short and long term. Based on the panel data of FDI flowing from 20 OECD countries to 52 host countries from 1996 to 2003, Barassi and Zhou [51] analyzed the impact of corruption on FDI, using both parametric and non-parametric methods. After controlling the location decisions of multinational enterprises and other characteristics of host countries, the results of both parametric and non-parametric methods supported the “grease the wheels” hypothesis. Using data of 14 developing countries, Subasat and Bellos [52] found that corruption helped FDI by shortening bureaucratic approval period, increasing government official efficiency, and evading regulation limitations. Fazira and Cahyadin [53] found that the higher a country ranked on the Corruption Perception Index (CPI) (i.e., less corruption in the country), the less FDI inflowed in six ASEAN member countries: Indonesia, Singapore, Malaysia, Thailand, the Philippines, and Vietnam.

Another branch is corruption and the entry mode of FDI. In corrupt host countries, foreign investors prefer to take on a local joint venture partner to cut through the bureaucratic maze. However, foreign investors with more sophisticated technologies are found to favor sole ownership [54].

### 2.4. Corruption and Economic Growth

As for the impact of corruption on economic growth, there are two opposed viewpoints in the existing studies: one is that corruption can promote economic growth. Entrepreneurs, by bribing officials, can reduce government intervention, simplify red tape in administrative approval, save time cost, improve the efficiency of government management, and avoid or delay the formulation of economic policy which is not conducive to the investment [55,56,57]. Some cases and empirical studies also support this viewpoint [58,59,60,61].

The other viewpoint is that corruption hinders economic growth. Murphy et al. [62] found that corruption would lead to misallocation of production factors and was bad for economic growth. Mauro [63] conducted an empirical study based on data of 58 countries and found that corruption would inhibit enterprise investment and economic growth. Mo [64] conducted an empirical study based on the panel data from 1970 to 1985 and found that corruption reduces the economic growth rate through three mechanisms: the first was the mechanism of human capital—that is, corruption encouraged entrepreneurs and workers to shift from production activities to rent-seeking activities, which would reduce production efficiency and output growth rate. The second was the mechanism of institution. Corruption was often accompanied by favoritism and cronyism, which led to political instability and reduction of the credibility of the government. The third was the mechanism of investment. Corruption reduced social investment and innovation, which in turn reduced economic growth. Blackburn et al. [65] found that corruption was not conducive to economic growth by constructing the dynamic general equilibrium model including corruption, tax revenue, and economic growth. Johnson et al. [66] studied the relationship between corruption and economic growth by using the panel data of American states from 1975 to 2007 and found that corruption was not conducive to economic growth. However, the extent of corruption’s impact on economic growth depended on the level of governmental regulation.

### 2.5. Research Gap

It seems that the impact of FDI on environmental pollution and the effect of corruption on FDI have both been well-documented in the extant literature. However, few previous studies have investigated the interaction effect between FDI and corruption on environmental pollution. In countries with serious corruption, low-quality, high-pollution FDI enter and engage in production by bribing local government officials, causing even more massive pollution emissions. The existing literature has shown that corruption affects the technology spillover effect of FDI. For example, the research of Smarzynska and Wei showed that, with the increase in corruption in the host countries, the tendency of the proprietorships’ investment of multinational enterprises increased significantly due to concern for judicial fairness and intellectual property protection. When the degree of corruption was high, the change in investment pattern and ownership structure was not conducive to FDI technology spillover [67]. Mauro found that the increase in corruption in the host country would significantly reduce the government’s expenditure in scientific research and public health, which was not conducive to the improvement of local scientific research and human capital. The technology spillover effect of FDI could only be generated effectively when the research and development (R&D) investment and human capital of the host country reached a certain level [68]. Therefore, if corruption is neglected, the impact of FDI on the environment will be erroneously estimated, and the corresponding environment protection policies will fail to achieve the desired results. To the best of the authors’ knowledge, Candau and Dienesch’s [69] is the only previous study that investigated this issue, and this study did not take into consideration the spatial factor in the environmental pollution in different regions. Wind direction and water currents may make the environment of one region susceptible to the pollution and emissions of the neighbor regions, and the geographic concentration of FDI intensifies the spatial autocorrelation of pollution [11,70,71]. Traditional panel data analysis neglects the impact of spatial correlation, possibly deriving biased results. This study attempts to address this research gap by applying the spatial econometric method to China’s provincial panel data.

## 3. Methodology and Data

### 3.1. The Econometric Model

To estimate the interaction effect between corruption and FDI on environmental pollution, this research proposes the following regression model:

(1) $$EP_{it}=\alpha_{0}+\alpha_{1}COR_{it}+\alpha_{2}FDI_{it}+\alpha_{3}COR_{it}\times FDI_{it}+\alpha_{4}{\vec{X}}_{it}+\lambda_{i}+\mu_{it}$$

In Equation (1), *i* and *t* stand for province and year, respectively. EP means environmental pollution index, COR represents official corruption, FDI is foreign direct investment. Therefore, $\alpha_{2}$ represents the direct influence of FDI on provincial environmental pollution. Because the relatively lower environmental regulation level in developing countries may be a reason that high-pollution corporations from developed countries relocate to developing countries, $\alpha_{2}$ is expected to be statistically significant and positive. COR×FDI is the interaction effect; therefore, $\alpha_{3}$ measures the degree to which the interaction effect influences environmental pollution. It seems plausible that the more serious corruption is, the larger the number of low-quality, high-pollution corporations that enter the country by bribing government officials to tolerate lower environmental standards. Therefore, $\alpha_{3}$ is expected to be statistically significant and positive. $\vec{X}$ is the vector of the control variables which may influence emissions. $\lambda_{i}$ is the provincial fixed effect. $\mu_{it}$ is the random disturbance term.

The wind blowing and the water flowing may make the environmental quality of one region susceptible to that of the neighbor regions, and the geographical clustering of FDI can enhance the spatial autocorrelation of pollution [70]. According to the different impact modes of the spatial correlation of the observed values, the commonly used spatial econometric models are the spatial lag model (SLM) and spatial errors model (SEM) [72].

The spatial lag model hypothesizes that the spatial correlation generates from the dependent variable and measures the influence of the environmental quality of the neighbor regions on that of the region in focus. Its econometric equation is as follows:

(2) $$EP_{it}=\rho W\times EP_{it}+\alpha_{0}+\alpha_{1}COR_{it}+\alpha_{2}FDI_{it}+\alpha_{3}COR_{it}\times FDI_{it}+\alpha_{4}{\vec{X}}_{it}+\lambda_{i}+\mu_{it}$$

In this above equation, W is the spatial weight matrix, and ρ is the spatial regression coefficient that reflects the impact of pollution in neighbor regions on that in the focused region.

The spatial errors model hypothesizes that spatial correlation generates from the error impact of the dependent variable and measures how the spillover effect caused by the error of the dependent variable for the neighbor regions influences the dependent variable for the region in focus. Its econometric model is as follows:

(3) $$\{\begin{array}{c} EP_{it}=\alpha_{0}+\alpha_{1}COR_{it}+\alpha_{2}FDI_{it}+\alpha_{3}COR_{it}\times FDI_{it}+\alpha_{4}{\vec{X}}_{it}+\lambda_{i}+\mu_{it}\\ \mu_{it}=\delta W\mu_{jt}+\eta_{it} \end{array}$$

In this above equation, δ is the spatial error coefficient. $\eta_{it}$ is the random disturbance term.

### 3.2. Variable Definitions and Data Sources

Because the environmental pollution in China mainly originates from industries, many previous studies use one or several of the three industrial wastes (waste gas, wastewater and solid waste) to be the measurement indicators of China’s environmental pollution (EP) [72,73,74,75]. In addition, China’s open-source statistical survey provides detailed and consistent data. Therefore, we adopt per capita industrial waste gas emissions (EP1) and per capita industrial wastewater emissions (EP2) as measurement indicators of industrial pollution.

COR represents the degree of corruption. In China, many corruption cases are highly implicit. Therefore, it is difficult to measure the exact degree of corruption. In cross-border comparative research, most previous studies use the Corruption Perception Index (CPI) published by Transparency International and the International Country Risk Guide index (ICRG) publicized by PRS Group [76,77,78,79,80,81]. However, such indicators are inapplicable to inter-regional studies within one country. When investigating the association between corruption and economic growth in different states of the USA, Glaeser and Saks [82] adopt the number of civil servants who are accused of power abuse to measure corruption. They assume that the number of corruption accusations is more accurate than survey results because accusation data have higher objectivity, longer time span, and are free from sampling errors and no-reply respondents. This research also adopts this method. The China Procuritorial Yearbook reveals the number of crime cases including embezzlement, bribery, malfeasance, and tort actions that provincial procuritorial authorities investigate annually, and this research adopts this number to represent the degree of corruption in China’s provinces, which is in accordance with most previous studies that probe China’s inter-regional corruption issues [75,83,84,85,86]. To exclude the effect of the scale of government and total population of the region, this study adopts the proportion of the number of duty crime cases in the number of public officials (COR1) and the proportion of the number of duty crime cases in total population (COR2) to be the measurement indicators of corruption.

Foreign direct investment (FDI) is represented by the ratio of actually utilized FDI in the region’s gross domestic product (GDP), according to the existing literature [9,18,87]. It is obtained by converting the US dollar value of FDI of the year into Chinese RMB with the average annual exchange rate and then dividing it by the GDP of the region.

W is the spatial weight matrix, which measures the degree of social and economic relationship between different provinces. Suppose the distance between regions *i* and *j* is $w_{ij}$, then the spatial weight matrix is as follows:

(4) $$W=\left(\begin{array}{ccc} w_{11} & \ldots & w_{1n}\\ \vdots & \ddots & \vdots \\ w_{n1} & \cdots & w_{nn} \end{array}\right)$$

W is an n×n symmetric matrix, and diagonal elements $w_{11}=\cdots =w_{nn}=0$, i.e., the distance between one region and itself is 0. The most frequently used spatial matrix in the existing research is the geographic adjacency matrix (a.k.a. 0–1 matrix). $w_{ij}=1$ when regions *i* and *j* are spatially adjacent, and $w_{ij}=0$ when the two regions are non-adjacent. It can be found that the geographic adjacency matrix assumes that spatial correlation exists only when two regions are geographically adjacent, while non-adjacent regions do not have spatial correlation. Therefore, a limitation may exist when measuring the spatial correlation between regions. We use three kinds of weight matrix: geographic distance weight matrix $W_{D}$, economic weight matrix $W_{E}$, and mixed weight matrix $W_{M}$ [88,89,90,91,92]. For the geographic distance weight matrix $W_{D}$, the European distance between the capitals of two provinces is calculated and normalized according to the latitude and longitude data of each provincial capital city. For the economic weight matrix $W_{E}$, the calculation formula for matrix element $w_{ij}$ is $w_{ij}=1/\left|GDP_{i}-GDP_{j}\right|S_{i}$, in which GDP_i_ and GDP_j_ are per capita GDP in province i and j, respectively, and $S_{i}=\sum_{j}\left(1/\left|GDP_{i}-GDP_{j}\right|\right)$ means the sum of the differences in the per capita GDP of the two provinces. The economic weight matrix $W_{E}$ implies that the environmental quality of provinces with similar economic development level may have strong spatial correlation. The mixed weight matrix $W_{M}=W_{D}\times W_{E}$ is the combination of geographic distance weight matrix and economic weight matrix. In the specific analysis, the three kinds of weight matrix will be standardized first and then considered as the weight of spatial individuals.

$\vec{X}$ is the vector variable which is composed by control variables. Referring to Huang et al. [9], Chang and Hao [79], Arminen and Menegaki [80], and Sinha et al. [81], this study selects the following control variables. The first control variable is the economic development level (Y), which is represented by GDP per capita. The relationship between economic development and environmental pollution is not linear: at the take-off stage, economic development leads to more pollution due to an imbalanced emphasis on rapid growth and negligence over environment protection. At higher economic development stages, the deteriorating environment, the increased environmental awareness of the public, the sophistication of environmental governance technologies, and the abundance of financial resources make economic development beneficial for environment quality. That is, the relationship between economic development and environmental pollution may be inverted U-shaped, as described by the environmental Kuznets curve (EKC) hypothesis in the extant literature [93,94,95]. Therefore, this study added the quadratic term of the economic development level (Y) to test whether the environmental Kuznets curve (EKC) hypothesis can be supported in the context of China’s regions. Moreover, the second control variable is industrial structure (IND), which is represented by the added value of secondary industries. Most high-pollution industries belong to the secondary industry; therefore, the higher the proportion of secondary industry in the economy, the heavier the pollution is. Therefore, the coefficient of this variable is anticipated to be positive. The third control variable is population density (POP), which is represented by the average population per unit area. The higher the population density, the greater the impact of human activities is on the natural environment. Accordingly, industrial corporations are usually more in the region. Therefore, this variable is anticipated to be positive in the equation. The fourth control variable is the degree of openness (OP) of the region, which is represented by the ratio of total value of import and export in GDP. To calculate, the annual import and export value in USD is first converted to RMB and then divided by GDP of the region. Openness on one hand facilitates the overall scale of related industries in the region of import and export businesses, i.e., increasing pollution in the region; on the other hand, trade with developed countries may encourage corporations in the region to adopt more advanced technologies and upgrade the industrial structure, i.e., decreasing pollution in the region. Therefore, the aggregate effect of openness on the environment remains uncertain. The fifth control variable is energy efficiency (EN), which is represented by the energy consumption per unit GDP. The increase in energy efficiency directly reduces the consumption of energy per unit of GDP, thus decreasing pollution. However, the rebound effect of energy efficiency may make the aggregate effect of energy efficiency on pollution uncertain. The sixth control variable is urbanization (UR), which is represented by the ratio of urban population in the total population. Previous literature suggests that urbanization usually consumes large amounts of natural resources and causes a lot of emissions; therefore, the coefficient of this variable is considered to be positive.

Considering the availability of China’s provincial data, this research takes panel data of 29 provinces from 1994 to 2015 as the sample. Data come from the China Statistical Yearbook, China Statistical Yearbook on Environment, China Compendium of Statistics, China Procuritorial Yearbook, China Labor Statistical Yearbook, China Statistical Yearbook for Regional Economy, and China Energy Statistical Yearbook.

## 4. Results and Discussions

### 4.1. Spatial Autocorrelation of Environmental Pollution in China’s Provinces

Before the spatial econometric analyses, it is necessary to test whether environmental pollution in different provinces has spatial autocorrelation. This research investigates the spatial concentration of environmental pollution in China from two perspectives: global spatial autocorrelation and local spatial autocorrelation. We analyze the global spatial autocorrelation with the global Moran’s I index proposed by Moran [96] and Geary’s C index proposed by Geary [97]. The formula of Moran’s I index is as follows:

(5) $$I=\frac{\sum_{i=1}^{n}\sum_{j=1}^{n}w_{ij}(z_{i}-\bar{z})(z_{j}-\bar{z})}{S^{2}\sum_{i=1}^{n}\sum_{j=1}^{n}w_{ij}}$$

In this equation, $z_{i}$ is the observed value of region *i*; n is the number of panels; S is the standard deviation of the sample; $w_{ij}$ is the element of spatial weight matrix, representing the distance between regions *i* and *j*. Moran’s I is the correlation coefficient between the observed value and its spatial lag term, the range of which is between −1 and 1. If Moran’s I is between 0 and 1, there is positive spatial autocorrelation. The closer the value is to 1, the stronger the positive spatial autocorrelation. If Moran’s I is between −1 and 0, there is negative spatial autocorrelation. The closer the value is to −1, the stronger the negative spatial autocorrelation. If Moran’s I is close to 0, it means random spatial distribution. The formula of Geary’s C index is as follows:

(6) $$C=\frac{(n-1)\sum_{i=1}^{n}\sum_{j=1}^{n}w_{ij}{\left(z_{i}-z_{j}\right)}^{2}}{2\left(\sum_{i=1}^{n}\sum_{j=1}^{n}w_{ij}\right)\left[\sum_{i=1}^{n}{\left(z_{i}-\bar{z}\right)}^{2}\right]}$$

Different from Moran’s I, the key part of Geary’s C is ${\left(z_{i}-z_{j}\right)}^{2}$. Therefore, the range of this index is between 0 and 2. If it is between 1 and 2, there is negative spatial autocorrelation, and if the index is between 0 and 1, there is positive spatial autocorrelation. If it is close to 1, it means random spatial distribution. Therefore, these two indexes behave in opposite directions. This research analyzes the spatial correlation of two pollutants (i.e., waste gas and wastewater) between 1994 and 2015 by applying Moran’s I, Geary’s C, and the geographic distance weight matrix $W_{D}$. See Table 1 for results.

From Table 1, it can be seen that both Moran’s I and Geary’s C indicate that per capita industrial waste gas and wastewater show positive and significant spatial correlation for all years at the 10% significance level at least. This finding suggests that, in the period of this research, China’s pollution shows spatial concentration, i.e., high-pollution regions are adjacent to each other, and low-pollution regions are adjacent to each other as well.

**Figure 1:**
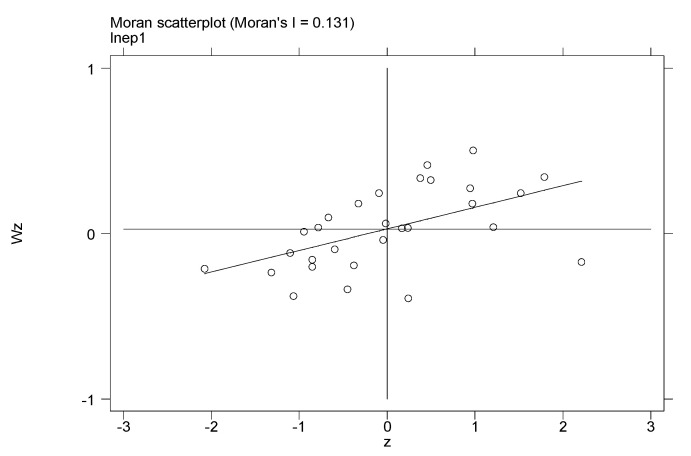
Moran’s I scatterplot for per capita industrial waste gas in 1994.

**Figure 2:**
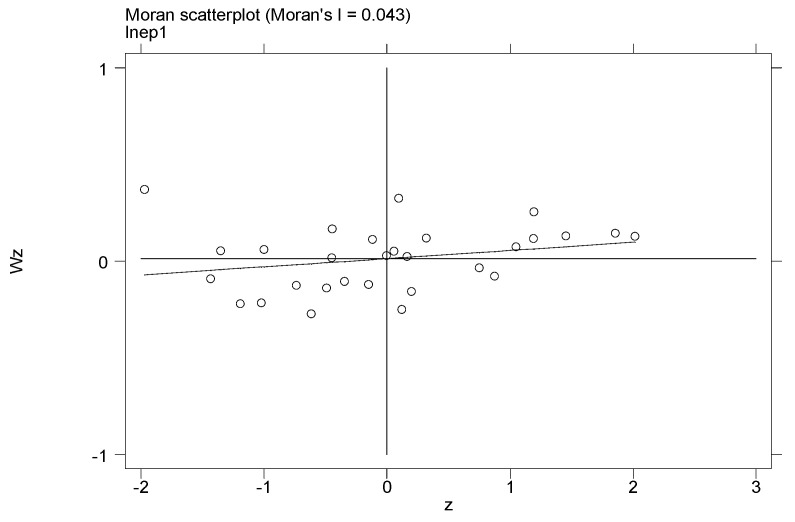
Moran’s I scatterplot for per capita industrial waste gas in 2015.

Moran’s I is more sensitive than Geary’s C in terms of local spatial autocorrelation; therefore, this research only uses local Moran’s I index and the corresponding scatterplot to show the local spatial autocorrelation of pollution. The scatterplot of Moran’s I index divides the pollution agglomeration of China’s provinces into four quadrants. Quadrant 1 means that a high-pollution region is adjacent to another high-pollution region (HH). Quadrant 3 means that a low-pollution region is adjacent to another low-pollution region (LL). Quadrants 2 (LH) and 4 (HL) mean that a high-pollution region is adjacent to a low-pollution region. Therefore, Quadrants 1 and 3 represent positive spatial autocorrelation, and Quadrants 2 and 4 represent negative spatial autocorrelation. This study calculates the local Moran’s I of EP1 (per capita industrial waste gas) for 1994 and 2015, and the corresponding scatterplots can be found in Figure 1 and Figure 2, in which the geographic distance weight matrix $W_{D}$ is used.

From Figure 1 and Figure 2, it can be seen that most scatterplots are in Quadrants 1 and 3. For 1994, 11 provinces are in Quadrant 1, namely Beijing, Tianjin, Hebei, Shanxi, Inner Mongolia, Liaoning, Jilin, Heilongjiang, Gansu, Ningxia, and Xinjiang. Moreover, 11 provinces are in Quadrant 3, namely Jiangsu, Anhui, Fujian, Jiangxi, Hubei, Hunan, Guangxi, Hainan, Sichuan, Guizhou, and Yunnan. In general, 22 provinces are in Quadrants 1 and 3, i.e., 75.8% of all the provinces in this study. For 2015, 10 provinces are in Quadrant 1, namely Hebei, Shanxi, Inner Mongolia, Shanghai, Anhui, Shandong, Gansu, Qinghai, Ningxia, and Xinjiang. Moreover, eight provinces are in Quadrant 3, namely Fujian, Jiangxi, Hubei, Hunan, Guangdong, Guangxi, Hainan, and Yunnan. In general, 18 provinces are in Quadrants 1 and 3, i.e., 62% of all the provinces in this study. This further shows that the environmental pollution in China’s provinces has significant positive spatial correlation, and pollution in one region will significantly affect the environmental quality of the adjacent regions.

### 4.2. Baseline Regression Results and Analyses

Although Moran’s I and Geary’s C indexes can help to justify the existence of the spatial correlation of pollution between China’s regions, they are unable to accurately estimate the degree of the correlation and the other influence factors of pollution. Therefore, we need further analyses based on Equations (2) and (3). Previous studies have shown that, compared with the ordinary least square (OLS) method, maximum likelihood estimation (MLE) is a better estimation method when estimating the parameters of the SEM or SLM model, because it can avoid possible endogeneity [90,98,99,100]. As for the application of SEM versus SLM, Anselin et al. [99] explained that, if LM lag is statistically more significant than LM error, while LM lag (robust) is significant and LM error (robust) is non-significant, the SLM model should be adopted. On the contrary, if LM error is statistically more significant than LM lag, and LM error (robust) is significant while LM lag (robust) is non-significant, the SEM model is more appropriate. According to this principle and the baseline regression results (see Table 2), it can be found that SLM is the appropriate model for this study.

In Table 2, columns (1–6) report the estimated effects of corruption and FDI on environmental pollution with data of all the 29 provinces in the sample. Columns (1–3) use per capita industrial waste gas (EP1) as the pollution index, and the spatial weight matrices are geographic distance, economic distance, and mixed weight matrix, respectively. Columns (4–6) use per capita industrial wastewater (EP2) as the pollution index, and accordingly, the spatial weight matrices are also geographic distance, economic distance, and mixed weight matrix, respectively.

It can be found that, in all the regression results, all spatial lag coefficients are significant and positive, indicating that pollution has a significant positive spatial effect between China’s regions, i.e., a high-pollution region is usually adjacent to other high-pollution regions, and a low-pollution region is also adjacent to other low-pollution regions, a finding that is consistent with the results of Moran’s I and Geary’s C indexes reported above. Corruption indicators are all positive and significant at the 5% significance level, indicating that official corruption will promote the industrial emissions. All FDI coefficients are significant and positive, and the differences between the equations are small, suggesting that the increase in FDI encourages pollution, a result that supports the PHH. In reality, due to higher levels of economic development and public awareness of environmental protection, environmental regulation is often more stringent, leading to higher expenses of pollution resolution and production costs. In contrast, due to lower levels of economic development, environmental regulation is less stringent in developing countries. In addition, some developing countries sacrifice environmental standards and regulations to attract FDI inflow and stimulate economic growth, i.e., “race to the bottom” [69,101]. Such differences give significant competitive advantage to developing countries in terms of policy and regulation and attract pollution intensive industries from developed countries to transfer their factories to developing countries through FDI.

The focus of this study is the interaction term between corruption and FDI. The coefficients of this interaction term are all positive and significant at the 5% significance level. The differences between the equations are small, suggesting that corruption not only encourages pollution directly by weakening the enforcement of environmental regulations but also causes pollution indirectly through the interaction effect between FDI and corruption. This may be due to the following two reasons. On one hand, corruption lowers the actual environmental regulation standards. When corruption is non-existent or at a low level, environmental regulation policies can be well executed, and only the FDI that fulfills environmental standards can enter and engage in production, while low-quality FDI is denied entry. When corruption is at a high level, the high-pollution FDI that would be denied by the nominal environmental standards can enter by bribing government officials and lowering the actual environmental standards [69,102]. The higher the corruption level, the greater the number of low-quality, high-pollution FDI corporations. The FDI that fulfills the nominal environmental regulation standards could also loosen the environmental monitoring by bribing government officials in order to use higher-pollution technologies for cost reduction, which all contribute to host country pollution. On the other hand, corruption affects the technology spillover of FDI. Previous research shows that FDI can enhance the level of production technology and pollution relief through the spillover effect, thus reducing the pollution of the host country. Firstly, compared to the domestic ones, FDI corporations usually possess more advanced technology and management experiences, which leads to a larger competitive advantage. Domestic corporations have to increase research and development expenditure in order to catch up (competition effect) or directly imitate the technology, marketing strategy, and management methods of FDI corporations (learning effect). Secondly, FDI employees have greater professional skills and will bring such skills and experiences to domestic corporations when they change their jobs or communicate with local company employees, thus improving the technology level of domestic businesses (mobility effect). Finally, FDI corporations help to improve domestic businesses that are in the upstream or downstream of their supply chain (vertical spillover effect). However, corruption seriously affects the spillover of FDI corporations and the learning process of domestic businesses, because FDI corporations tend to choose wholly owned subsidiaries over joint ventures as entry modes due to concerns about institutional environment and intellectual property rights, and high-technology corporations may also decrease their investment in the host country. When corruption significantly affects the day-to-day operations of FDI corporations, they may achieve their production through the direct acquisition of domestic businesses. This mode of investment will weaken the spillover effect of FDI corporations [39,67]. Domestic businesses need a certain degree of technology and human resources to absorb FDI spillover, but increased corruption levels will impede government expenditure in science and education and harm host country technology progress and human capital accumulation, weakening the learning ability of local firms.

Among the control variables, the first-order term coefficient of economic development (Y) is significant and positive, and the coefficient of the quadratic term of Y is significant and negative, indicating the inverted U-shaped association between economic development and pollution, i.e., the applicability of the environmental Kuznets curve in China. The coefficient of industrial structure (IND) is positive and significant at the 5% level, indicating that industrial structure is a key factor of pollution, and the higher the secondary industry in the national economy, the heavier the pollution is. This is because secondary industries, particularly the textile, paper, and chemical material product industries, are heavily polluting and harmful to the environment. The coefficient of population density (POP) is negative and significant at the 5% level. It is generally believed that higher population density leads to greater demand for products, and accordingly, the number of corporations and degree of pollution are higher. However, population density may also increase the pressure on the environment, and the public pursuit for environmental protection is stronger, thus inducing a reinforcing effect on environmental regulation, preventing the local environment from further deterioration. The coefficient of degree of openness (OP) is positive and significant at the 10% level, suggesting that outbound trade may harm the environment. Compared with developed countries, China has a comparative advantage in pollution intensive products, and foreign trade further increases the scale of such industries, increasing the pollution level. The coefficient of energy efficiency (EN) is significant at the 1% level, indicating that the increase in energy efficiency encourages pollution, a finding that is counterintuitive to common knowledge. It is generally believed that an increase in energy efficiency will directly reduce energy consumption per unit of GDP, thus reducing environmental pollution. However, increased energy efficiency means that the same amount of energy can produce more product, or the same product will cost less energy efficiency. Such changes will drive down the market price of energy as well as production costs. This way, producers and consumers will use even more products, a phenomenon known as the rebound effect [103,104]. If the increase in product use exceeds the savings thanks to increased energy efficiency, then higher energy efficiency leads to more pollution. The coefficient of urbanization is positive and significant at the 10% level, indicating that urbanization leads to pollution. This is because urban construction, resident activities (e.g., automobile exhaust), and the exothermic nature of direct and indirect human activities discharge large amounts of pollution.

### 4.3. Robustness Test

To ensure the robustness of the analysis, this study carries out several robust tests. Firstly, besides the geographic distance weight matrix, we also use two other matrices, namely economic distance weight and mixed weight. Both industrial waste gas and wastewater are adopted as indicators of environmental pollution to validate the robustness of the results for different weight matrices and pollution indicators (see in Table 2). From the analyses discussed above, for the same pollution indicator, coefficients of corruption, FDI, and the interaction term between corruption and FDI are all of the same direction in different weight matrices, suggesting that the results are robust. Secondly, besides the proportion of the number of duty crime cases in the number of public officials (COR1) to measure corruption, this study also includes the proportion of the number of duty crime cases in the total population (COR2) to be a proxy variable of corruption. The results can be seen in Table 3.

From Table 3, it can be seen that, when using the proportion of the number of duty crime cases in the total population (COR2) to be a proxy variable of corruption, the results of concerned explanatory variables in Table 3 are generally in accordance with those in Table 2, validating the robustness of the analysis.

### 4.4. Empirical Results and Analyses by Region

In this section, we divide the whole sample into eastern, central, and western regions and discuss the differentiated effects in these three sub-samples. The provinces in eastern, central, and western regions are shown in Table 4.

Econometric results can be found in Table 5, in which all models use the geographic distance matrix ($W_{D}$) to be the weight matrix.

In Table 5, the coefficients of corruption, FDI, and the interaction term between corruption and FDI are all positive and significant, a result in accordance with the whole-sample analysis results. Further comparison shows that, in the regression equations for waste gas and wastewater, the coefficients of the interaction term between corruption and FDI are all smaller in the eastern region than in the central and western regions, indicating that the interaction effect between corruption and FDI on pollution is lower in the eastern than in the central and western regions, which may be due to the following reasons. Firstly, per capita income in the eastern region is higher than in the central and western regions; therefore, the public environmental awareness is also higher in the east. The same degree of corruption causes less weakening effect on environmental regulation in the eastern region than in the central and western regions. That is, the environmental quality standard in the eastern region is higher than in the central and western regions. Low-quality, high-pollution FDI corporations are relatively less in the east, causing less pollution than in the central and western regions. Secondly, compared to the central and western regions, level of economic development and market sophistication is higher in the eastern region; therefore, FDI corporations with more advanced technology are more willing to invest in the east. At the same corruption level, FDI spillover effect is greater in the east. Moreover, the fiscal income of eastern governments is also higher; therefore, more financial resources are invested in research and development as well as education. Therefore, the R&D and human resource performance of domestic companies in the east is also higher, leading to better learning capacity for FDI spillover. In summary, at the same corruption level, FDI spillover is higher, while pollution is lower, in the eastern region than in the central and western regions.

## 5. Conclusions

This study investigates the effects of local government corruption and FDI on environmental pollution by applying the spatial econometric model to the panel data of China’s 29 provinces from 1994 to 2015 and analyzes the inter-regional differences among the eastern, central, and western regions. Results show that (a) the coefficients of FDI are always significant and positive irrespective of pollution being measured with per capita industrial waste gas or wastewater and irrespective of adopting geographic distance weight, economic distance weight, or mixed weight as the spatial weight matrix. This indicates that FDI inflow worsens China’s environmental quality, supporting the PHH. (b) The coefficients of the interaction term between corruption and FDI are all significant and positive, suggesting that corruption indirectly leads to pollution by lowering the actual environmental standards, enabling low-quality FDI inflow and weakening the FDI spillover effect. (c) The interaction effect between corruption and FDI on pollution is different across different regions, and the effect is less significant in the eastern region than in the central and western regions.

There are some limitations in this paper. Firstly, this paper uses the aggregate data at the provincial level in China. The research by Holz and Lin [105] showed that the definitions of variables and the classification of firms in China have changed frequently since the reform and opening-up. Therefore, future studies that use firm-level data should reach more accurate conclusions. However, to the best of our knowledge, there are no firm-level FDI inflow data in China. Secondly, the research of Godinez and Liu [106] showed that FDI was influenced by the difference in corruption level between the host and home countries, which was defined as “corruption distance”, rather than just the corruption level of the host country. FDI will be affected only when the corruption level of the home country is lower than that of the host country, i.e., the corruption distance is negative. Inspired by their research, the corruption level of both host and home countries of FDI should be taken into account simultaneously when studying the interaction of corruption and FDI on environmental pollution in the future research.

## 6. Policy Implications

According to the Corruption Perception Index, China has ranked around 80th in all the countries counted in the last five years. In particular, China dropped to 100th in 2014, indicating that corruption is still rampant. As the conclusion of this research suggests, restricting FDI or curbing the corruption of local government officials will help to decrease environmental pollution. However, FDI plays a key role in promoting China’s economic growth; therefore, a restriction on FDI exclusively for pollution reduction may not be a good option. Therefore, a major policy improvement may come from monitoring local government officials and curbing corruption levels. Specifically, (a) central government authorities should strengthen their supervision of government departments. Supervision and power constraints should also be strengthened within the political party in office. The division between fiscal and executive powers of central and local governments should be crystalized. Independence of jurisdiction should be guaranteed institutionally in order to prevent local governments from interfering or directly administering judicial authorities. Government functions should be improved so that government approval processes can be simplified to lower the possibility of corruption. (b) The cost of government corruption must be raised, including the psychological cost, opportunity cost, and the punishment measures after investigations. Psychological and opportunity costs of corruption may be difficult to alter. Therefore, the key to raise the cost of corruption is to increase the likelihood of investigation and the scale of punishment—that is, a larger judicial cadre team, a broader and heavier strike against administrative corruption, and more serious punishment to government officials sentenced for proven corrupt behaviors. (c) Establish public and social media monitoring channels. Only when different monitoring channels work together can corruption be reduced to the minimum level. Finally, the technological threshold of FDI entry should be gradually increased in order to filter out low-quality FDI. Government expenditure on education and R&D should also be increased to improve the human capital and learning capacity of domestic businesses.

## Figures and Tables

**Table 1:** Moran’s I index and Geary’s C index under the geographic distance weight matrix for EP1 and EP2.

Year	EP1		EP2	
	Moran’s I	Geary’s C	Moran’s I	Geary’s C
1994	0.131 ***	0.832 ***	0.158 ***	0.820 ***
1995	0.140 ***	0.820 ***	0.149 **	0.834 ***
1996	0.128 ***	0.841 ***	0.163 ***	0.909 ***
1997	0.156 ***	0.822 ***	0.142 **	0.832 ***
1998	0.130 ***	0.850 ***	0.137 **	0.841 ***
1999	0.147 ***	0.871 ***	0.150 **	0.819 **
2000	0.109 ***	0.873 ***	0.162 ***	0.800 **
2001	0.127 ***	0.866 ***	0.173 ***	0.804 **
2002	0.126 ***	0.868 ***	0.165 ***	0.805 **
2003	0.136 ***	0.856 ***	0.047 **	0.817 *
2004	0.096 ***	0.883 ***	0.043 **	0.821 ***
2005	0.126 ***	0.856 ***	0.124 ***	0.883 ***
2006	0.123 ***	0.859 ***	0.105 ***	0.866 ***
2007	0.114 ***	0.820 ***	0.121 ***	0.872 ***
2008	0.092 ***	0.819 ***	0.128 ***	0.883 ***
2009	0.110 ***	0.818 ***	0.115 ***	0.875 ***
2010	0.165 ***	0.853 **	0.132 ***	0.895 ***
2011	0.158 **	0.828 *	0.105 ***	0.870 ***
2012	0.131 **	0.838 **	0.107 ***	0.881 ***
2013	0.122 **	0.853 **	0.121 ***	0.864 ***
2014	0.127 **	0.833 **	0.113 ***	0.879 ***
2015	0.128 **	0.832 **	0.124 ***	0.877 ***

Note: ***, **, and * mean significant at 1%, 5%, 10% levels.

**Table 2:** Baseline regression results.

Explanatory Variables	EP1			EP2		
	(1)	(2)	(3)	(4)	(5)	(6)
COR1	0.03 **	0.03 **	0.02 **	0.10 **	0.19 ***	0.18 ***
	(0.04)	(0.04)	(0.04)	(0.05)	(0.05)	(0.05)
FDI	0.01 **	0.01 **	0.01 **	0.06 **	0.08 ***	0.08 ***
	(0.02)	(0.02)	(0.02)	(0.02)	(0.02)	(0.02)
COR1×FDI	0.01 **	0.01 **	0.01 **	0.01 **	0.02 ***	0.02 ***
	(0.01)	(0.01)	(0.01)	(0.01)	(0.01)	(0.01)
Y	1.62 ***	1.61 ***	1.65 ***	0.51 *	0.25 **	0.33 **
	(0.24)	(0.22)	(0.23)	(0.16)	(0.16)	(0.16)
Y^2^	−0.04 ***	−0.04 ***	−0.04 ***	−0.03 ***	−0.02 ***	−0.03 ***
	(0.01)	(0.01)	(0.01)	(0.04)	(0.04)	(0.04)
IND	0.01 ***	0.01 ***	0.01 ***	0.01 ***	0.01 **	0.01 ***
	(0.01)	(0.01)	(0.01)	(0.02)	(0.01)	(0.01)
POP	−0.47 ***	−0.37 **	−0.32 **	−0.77 ***	−0.60 ***	−0.65 ***
	(0.15)	(0.14)	(0.14)	(0.18)	(0.18)	(0.18)
OP	0.01 **	0.01 **	0.01 ***	0.01 **	0.01 **	0.01 *
	(0.01)	(0.01)	(0.01)	(0.01)	(0.01)	(0.01)
EN	0.73 ***	0.73 ***	0.73 ***	0.87 ***	0.77 ***	0.79 ***
	(0.05)	(0.05)	(0.05)	(0.07)	(0.07)	(0.07)
UR	0.02 ***	0.02 ***	0.02 ***	0.01 **	0.01 *	0.01 *
	(0.01)	(0.01)	(0.01)	(0.01)	(0.01)	(0.01)
ρ	0.23 ***	0.20 ***	0.15 ***	0.63 ***	0.17 ***	0.05 *
	(0.05)	(0.04)	(0.03)	(0.11)	(0.06)	(0.05)
R^2^	0.86	0.77	0.76	0.91	0.86	0.83
LM Lag	359 ***	15 ***	34.9 ***	5.45 **	4.61 **	3.32 **
LM Lag(Robust)	357 ***	15 ***	34.8 ***	5.51 **	4.56 **	3.28 **
LM Error	1.91	0.05	0.11	0.01	0.05	0.04
LM Error(Robust)	0.17	0.01	0.01	0.07	0.01	0.01
Weight Type	W_D_	W_E_	W_M_	W_D_	W_E_	W_M_

Note: ***, **, and * mean significant at 1%, 5%, 10% levels; standard errors in parentheses.

**Table 3:** Robustness test: alternative indicators of corruption.

Explanatory Variables	EP1			EP2		
	(1)	(2)	(3)	(4)	(5)	(6)
COR2	0.06 **	0.05 **	0.05 **	0.07 **	0.15 ***	0.14 **
	(0.04)	(0.04)	(0.04)	(0.05)	(0.05)	(0.05)
FDI	0.01 ***	0.01 ***	0.01 ***	0.01 ***	0.01 ***	0.01 ***
	(0.02)	(0.02)	(0.02)	(0.02)	(0.02)	(0.02)
COR2×FDI	0.01 **	0.01 **	0.01 **	0.01 **	0.01 **	0.01 **
	(0.01)	(0.01)	(0.01)	(0.01)	(0.01)	(0.01)
Control Variables	Yes	Yes	Yes	Yes	Yes	Yes
ρ	0.23 ***	0.19 ***	0.15 ***	0.65 ***	0.15 **	0.03 *
	(0.05)	(0.04)	(0.03)	(0.11)	(0.06)	(0.05)
R^2^	0.74	0.76	0.72	0.90	0.86	0.82
Weight Type	W_D_	W_E_	W_M_	W_D_	W_E_	W_M_

Note: ***, **, and * mean significant at 1%, 5%, 10% levels; standard errors in parentheses.

**Table 4:** The provinces in eastern, central, and western regions.

Region	Province
Eastern	Beijing, Tianjin, Hebei, Liaoning, Shanghai, Jiangsu, Zhejiang, Fujian, Shandong, Guangdong, and Hainan
Central	Shanxi, Jilin, Heilongjiang, Anhui, Jiangxi, Henan, Hubei, and Hunan
Western	Inner Mongolia, Guangxi, Sichuan, Guizhou, Yunnan, Shaanxi, Gansu, Qinghai, Ningxia, and Xinjiang

**Table 5:** Empirical results of different regions.

Explanatory Variables	Eastern		Central		Western	
	EP1	EP2	EP1	EP2	EP1	EP2
	(1)	(2)	(3)	(4)	(5)	(6)
COR1	0.05 *	0.14 **	0.09 *	0.35 ***	0.11 **	0.18 *
	(0.07)	(0.09)	(0.11)	(0.12)	(0.09)	(0.11)
FDI	0.06 **	0.05 *	0.17 ***	0.41 ***	0.17 ***	0.19 ***
	(0.02)	(0.03)	(0.13)	(0.15)	(0.15)	(0.18)
COR1×FDI	0.01 **	0.01 *	0.04 ***	0.10 ***	0.05 ***	0.06 ***
	(0.01)	(0.01)	(0.03)	(0.04)	(0.04)	(0.05)
Control Variables	Yes	Yes	Yes	Yes	Yes	Yes
ρ	0.08 *	0.52 ***	0.29 ***	0.04 *	0.26 ***	0.43 ***
	(0.07)	(0.11)	(0.08)	(0.11)	(0.08)	(0.13)
R^2^	0.77	0.77	0.73	0.80	0.83	0.87
Weight Type	W_D_	W_E_	W_M_	W_D_	W_E_	W_M_

Note: ***, **, and * mean significant at 1%, 5%, 10% levels; standard errors in parentheses.
